# Link between cognitive impairment and metabolic syndrome in middle-aged patients

**DOI:** 10.1192/j.eurpsy.2021.666

**Published:** 2021-08-13

**Authors:** N. Neznanov, V. Piotrovskaya, N. Burmistrova, A. Tabulina

**Affiliations:** Psychiatry And Narcology, FSBI First Pavlov Medical Univercity, Sankt Petersburg, Russian Federation

**Keywords:** Metabolic syndrome, mild cognitive impairment, vascular disorders, dementia

## Abstract

**Introduction:**

Metabolic syndrome (MS) is associated with an increased risk of developing a cognitive vascular disorders and dementia.

**Objectives:**

The associations of cognitive disorders (CD) with components of methabolic syndrome (MS) such as : body mass index, lipid spectrum, arterial hypertension and glucose level (GL) in middle age subjects were study.

**Methods:**

The 271 patients with MS according IDF criteria, (aged 30 – 60 years) were examend. Current mild cognitive impairment (MCI) was confirmed by psychodiagnostic interview according to the criteria of ICD-10. All patients passed through: MMSE test, Cognitive Failures Questionnaire, Wechsler memory scale, Symbol Coding and Category Fluency test. Level of blood glucose and plasma indicators of lipid spectrum were assessed in the blood samples with «Abbott» kits. To assess the results the NCEP criteria were used.

**Results:**

All 271 subject were divided into 2 groups, group A – with CD and/or MCI (212 subjects) and the group B -without affective disorders (49 subjects). Using the Mann-Whitney test significantly strong connection between high levels of total cholesterol (TC), cholesterol low density lipoprotein (LDL-C), lipoproteins of very low density (VLDL), the GL and MCI in group A were obtained. Optional subjects with sings of PH, MS and MCI had a fairly high level of VLDL and LDL-C in comparison with subjects without MCI.

**Conclusions:**

The meaning of the relationship between metabolic syndrome and mild cognitive impairments in middle-aged people is in increasing in the level of LDL and VLDL that can provoke MCI in middleage subjects with MS.

